# Estradiol Increases Glutamate and GABA Neurotransmission into GnRH Neurons via Retrograde NO-Signaling in Proestrous Mice during the Positive Estradiol Feedback Period

**DOI:** 10.1523/ENEURO.0057-18.2018

**Published:** 2018-08-03

**Authors:** Imre Farkas, Flóra Bálint, Erzsébet Farkas, Csaba Vastagh, Csaba Fekete, Zsolt Liposits

**Affiliations:** 1 Laboratory of Endocrine Neurobiology, Institute of Experimental Medicine, Hungarian Academy of Sciences, Budapest H-1083, Hungary; 2 Laboratory of Reproductive Neurobiology, Institute of Experimental Medicine, Hungarian Academy of Sciences, Budapest H-1083, Hungary; 3 Roska Tamás Doctoral School of Sciences and Technology, Faculty of Information Technology and Bionics, Pázmány Péter Catholic University, Budapest H-1083, Hungary; 4 Laboratory of Integrative Neuroendocrinology, Institute of Experimental Medicine, Hungarian Academy of Sciences, Budapest H-1083, Hungary; 5 Division of Endocrinology, Diabetes and Metabolism, Department of Medicine, Tupper Research Institute, Tufts Medical Center, Boston, MA 02111; 6 Department of Neuroscience, Faculty of Information Technology and Bionics, Pázmány Péter Catholic University, Budapest H-1083, Hungary

**Keywords:** GABA, glutamate, GnRH neuron, positive estradiol feedback, retrograde nitric oxide signalling, slice electrophysiology

## Abstract

Surge release of gonadotropin-releasing hormone (GnRH) is essential in the activation of pituitary gonadal unit at proestrus afternoon preceded by the rise of serum 17β-estradiol (E2) level during positive feedback period. Here, we describe a mechanism of positive estradiol feedback regulation acting directly on GnRH-green fluorescent protein (GFP) neurons of mice. Whole-cell clamp and loose patch recordings revealed that a high physiological dose of estradiol (200 pM), significantly increased firing rate at proestrus afternoon. The mPSC frequency at proestrus afternoon also increased, whereas it decreased at metestrus afternoon and had no effect at proestrus morning. Inhibition of the estrogen receptor β (ERβ), intracellular blockade of the Src kinase and phosphatidylinositol 3 kinase (PI3K) and scavenge of nitric oxide (NO) inside GnRH neurons prevented the facilitatory estradiol effect indicating involvement of the ERβ/Src/PI3K/Akt/nNOS pathway in this fast, direct stimulatory effect. Immunohistochemistry localized soluble guanylate cyclase, the main NO receptor, in both glutamatergic and GABAergic terminals innervating GnRH neurons. Accordingly, estradiol facilitated neurotransmissions to GnRH neurons via both GABA_A_-R and glutamate/AMPA/kainate-R. These results indicate that estradiol acts directly on GnRH neurons via the ERβ/Akt/nNOS pathway at proestrus afternoon generating NO that retrogradely accelerates GABA and glutamate release from the presynaptic terminals contacting GnRH neurons. The newly explored mechanism might contribute to the regulation of the GnRH surge, a fundamental prerequisite of the ovulation.

## Significance Statement

One of the major reasons of infertility is the failure in ovulation. Therefore, understanding of the neuronal processes resulting in proper ovulation is indispensable. Although hypothalamic gonadotropin-releasing hormone (GnRH) neurons are regarded the master-neurons orchestrating reproduction, most of the relevant papers have suggested so far that the estradiol positive feedback (indispensable for ovulation) acts in these neurons mostly indirectly, via hypothalamic nuclei free of GnRH neurons. Now we have presented strong evidence that estradiol exerts fast direct stimulatory action in GnRH neurons during the positive feedback, by activating retrograde nitric oxide (NO) signaling to accelerate excitatory synaptic inputs to GnRH neurons. In addition, we suggest a putative mechanism, whereby indirect and direct actions of estradiol can work in concert regulating GnRH neurons.

## Introduction

Reproduction is sustained by the accurate physiologic performance of the hypothalamo-pituitary-gonadal (HPG) axis (Knobil, 1988; Berga and Naftolin, 2012). The different constituents of the axis form a regulatory loop and communicate with each other via hormone messengers. The descending regulatory cascade of the loop utilizes gonadotropin-releasing hormone (GnRH) released in pulsatile manner (Belchetz et al., 1978; Clarke et al., 1987) from the hypothalamus and two gonadotrophic hormones, the luteinizing hormone (LH) and the follicle-stimulating hormone (FSH), discharged from the anterior pituitary (Knobil, 1988). The trophic effects of GnRH, LH, and FSH maintain gametogenesis and hormone production of the gonads. In females, the gonadal steroid hormones, 17β-estradiol (E2) and progesterone, form the ascending wing of the loop that informs the brain about the actual gonadal steroid milieu at the periphery. The brain senses the progression of the gonadal cycle (Couzinet and Schaison, 1993; Berga and Naftolin, 2012) by monitoring the estrous cycle-dependent changes in blood E2 level (Nelson et al., 1992; Freeman, 1994; Christian et al., 2005; Chu et al., 2009) and shows characteristic responses to both negative (low E2) and positive (high E2) feedback effects (Caraty et al., 1995; Moenter et al., 2009; Christian and Moenter, 2010; Radovick et al., 2012). Estradiol signals reach both the GnRH master neurons (Hrabovszky et al., 2000, 2001, 2007; Kenealy and Terasawa, 2012) and a wide scale of neuron assemblies connected with GnRH cells via synapses (Gore, 2001; Moenter, 2010; Cheong et al., 2015; Adams et al., 2018). The fact that rodent GnRH neurons express estrogen receptors (ERs; Hrabovszky et al., 2000, 2001), particularly the β-subtype, makes the scenario of their direct targeting by the E2 feedback conceivable. Indeed, E2 can directly influence GnRH neurons of female rodents (Cheong et al., 2012) suppressing various functions of these cells during the negative E2 feedback period (Kwakowsky et al., 2012; Balint et al., 2016). This negative feedback switches to positive estradiol feedback in the late afternoon of proestrus which switch is indispensable in the induction of surge release of GnRH eventually leading to the ovulation (Arimura et al., 1974; Moenter et al., 1990, 2009; Caraty et al., 1995; Christian and Moenter, 2010; Radovick et al., 2012). The role of the medial preoptic area (POA), the anteroventral periventricular nucleus (AVPV) and the arcuate nucleus (nARC) in mediating effects of E2 during positive feedback period and preparation of GnRH neurons for the surge release has been extensively studied (Ottem et al., 2004; Chakraborty et al., 2005; Smith et al., 2005, 2006; Bodo et al., 2006; Popa et al., 2008; Dungan Lemko et al., 2010; Porteous et al., 2011; Hrabovszky et al., 2012b; Liu and Herbison, 2013; Chassard et al., 2015; Dubois et al., 2015; Mittelman-Smith et al., 2016; Wang et al., 2016; Adams et al., 2018). In contrast, our current knowledge about direct targeting of mouse GnRH neurons by the positive E2 feedback in the proestrous phase of the ovarian cycle is limited (Farkas et al., 2013; Silveira et al., 2017). To address this issue, patch clamp electrophysiology and immunocytochemistry were used to study the putative role of ERβ and the significance of nitric oxide (NO) signaling, a recently discovered regulatory mechanism operating in GnRH neurons (Farkas et al., 2016; Bedenbaugh et al., 2018), in the mediation and execution of the positive E2 feedback effect directly in GnRH neurons.

## Materials and Methods

### Ethics statement

All studies were carried in accordance with legal requirements of the European Community. All animal experimentation described was conducted in accord with accepted standards of humane animal care and all efforts were made to minimize suffering.

### Experimental animals

Adult, gonadally intact GnRH-green fluorescent protein (GnRH-GFP) transgenic female mice with C57BL/6J genetic background were used for electrophysiological experiments. In this animal model, a GnRH promoter segment drives selective GFP expression in the majority of GnRH neurons (Suter et al., 2000). Phase of the estrous cycle was determined by both evaluating vaginal smears (Nelson et al., 1982; Caligioni, 2009; Byers et al., 2012) and visual observation of the vaginal opening using the method elaborated recently (Caligioni, 2009; Byers et al., 2012). In addition, mass of uterus was measured and mice having an uterus beyond 100 mg were considered as proestrous ones, whereas animals with uterus mass below 80 mg were regarded as metestrous ones (Silveira et al., 2017). Mice were maintained in 12/12 h light/dark cycle (lights on at 6 A.M.) and temperature controlled environment (22 ± 2°C), with standard rodent chow and tap water available *ad libitum*. All mice were housed in the same room under same environmental conditions.

### Brain slice preparation and recording

Mice were deeply anesthetized by isoflurane inhalation just before termination. They were killed either in the morning between 9 and 10 A.M. and the related recordings started at 11 A.M. (experiments at the morning of proestrus) or in the afternoon between 4 and 5 P.M., and the related recordings started at 6 P.M. (experiments at the afternoon of proestrus or metestrus). After decapitation, the brain was removed rapidly and immersed in ice-cold cutting solution extensively bubbled with a mixture of 95% O_2_ and 5% CO_2_ (O_2_/CO_2_). The cutting solution contained the following: 205 mM saccharose, 2.5 mM KCl, 26 mM NaHCO_3_, 5 mM MgCl_2_, 1.25 mM NaH_2_PO_4_, 1 mM CaCl_2_, and 10 mM glucose. Forebrain blocks were dissected and 250-μm-thick coronal slices were prepared from the medial septum/POA with a VT-1000S Vibratome (Leica GmbH) and placed in the ice-cold oxygenated cutting solution. The slices containing POA were transferred into artificial CSF (aCSF; 130 mM NaCl, 3.5 mM KCl, 1.25 mM NaH_2_PO_4_, 1.2 mM MgSO_4_, 2.5 mM CaCl_2_, 26 mM NaHCO_3_, and 10 mM glucose) saturated with O_2_/CO_2_ and kept in it for 1 h to equilibrate. Equilibration started at 33°C, and the aCSF was allowed to cool to room temperature. Mass of uterus was measured after brain cutting and slices from metestrous mice having uterus beyond 80 mg, and those from proestrous mice below 100 mg were discarded (Silveira et al., 2017).

Electrophysiological recordings were conducted at 33°C in aCSF bubbled with O_2_/CO_2_. Axopatch 200B patch clamp amplifier, Digidata-1322A data acquisition system, and pCLAMP 10.4 software (Molecular Devices Co.) were used for recording. Cells were visualized with a BX51WI IR-DIC microscope (Olympus Co.) located on an anti-vibration table (Supertech Kft). The patch electrodes (OD = 1.5 mm, thin wall; Hilgenberg GmbH) were pulled with a Flaming-Brown P-97 puller (Sutter Instrument Co., Novato, CA) and polished with an MF-830 microforge (Narishige). GnRH-GFP neurons were identified by brief illumination at 470 nm using an epifluorescent filter set, based on their green fluorescence, typical fusiform shape, and topographic location in the POA (Suter et al., 2000).

### Whole-cell patch clamp experiments

The cells were voltage clamped at -70-mV holding potential. Pipette offset potential, series resistance (R_s_) and capacitance were compensated before recording. Only cells with low holding current (<≈10 pA) and stable baseline were used. Input resistance (R_in_), R_s_, and membrane capacitance (C_m_) were also measured before each recording by using 5-mV hyperpolarizing pulses. To ensure consistent recording qualities, only cells with R_s_ < 20 MΩ, R_in_ > 500 MΩ, and C_m_ > 10 pF were accepted.

There is a widely accepted consensus that GABA is excitatory via GABA_A_-R in GnRH neurons due to the high intracellular chloride concentration in these cells (DeFazio et al., 2002; Moenter and DeFazio, 2005; Herbison and Moenter, 2011). Our main goal was to mimic the physiologic conditions of GnRH neurons as much as possible during the entire experiment, therefore, high concentration of chloride in the intracellular solution was indispensable. According to this requirement, the intracellular pipette solution contained high amount of chloride: 10 mM HEPES, 140 mM KCl, 5 mM EGTA, 0.1 mM CaCl_2_, 4 mM Mg-ATP, and 0.4 mM Na-GTP (pH 7.3). The resistance of the patch electrodes was 2–3 MΩ. For the miniature PSC (mPSC) measurements, 10 min before the start of recording, the spike-mediated transmitter release was blocked by adding the voltage sensitive Na-channel inhibitor tetrodotoxin (TTX; 646 nM; Tocris) to the aCSF. In the experiments, where the involved neurotransmitter receptors were examined, the GABA_A_-Rs were blocked by picrotoxin (100 μM), the ionotropic glutamate-receptors (Glu-Rs) were inhibited by the Glu-R inhibitor kynurenic acid (2 mM), and the AMPA/kainate-Rs were antagonised by the AMPA/kainate-R antagonist NBQX (10 μM), added to the aCSF during the respective recordings. To demonstrate the effect of E2, the recordings were conducted with an initial control recording (5 min), then high physiologic dose of E2 (200 pM, Sigma), characteristic for the proestrous phase of the estrous cycle in rodents (Nelson et al., 1992; Christian et al., 2005; Freeman, 2006; Chu et al., 2009), was added to the aCSF in the recording chamber and the recording continued for a subsequent 10 min. Stock of E2 (10 mM) was prepared in DMSO, which was diluted in aCSF to reach the final working concentration (200 pM) pipetted onto the slice in a single bolus. Instantaneous volume of the aCSF in the chamber was 1.5 ml. A single bolus of 3 µl of 100 nM E2 (diluted in aCSF) was pipetted to the inlet of the chamber. Using these volume and concentration data, 3 µl of 100 nM E2 in 1.5 ml aCSF gives the actual dose of E2 (200 pM) in the chamber. Flow rate of perfusion was 3 ml/min. When the ERβ antagonist 4-[2-phenyl-5,7-bis(trifluoromethyl)pyrazolo[1,5-a]pyrimidin-3-yl]phenol (PHTPP; 1 μM) was used extracellularly, it was added to the aCSF 10 min before starting the recording, whereas in case of its intracellular application (intraPHTPP) the drug was added to the intracellular solution in the pipette. The membrane-impermeable NO-scavenger 2-(4-carboxyphenyl)-4,4,5,5-tetramethylimidazoline-1-oxyl-3-oxide (CPTIO; 1 mM), the Src kinase inhibitor 4-amino-3-(4-chlorophenyl)-1-(t-butyl)-1H-pyrazolo[3,4-d]pyrimidine,4-amino-5-(4-chlorophenyl)-7-(t-butyl)pyrazolo[3,4-]pyrimidine (PP2; 10 µM) or the phosphatidylinositol 3 kinase (PI3K) blocker 2-(4-morpholinyl)-8-phenyl-1(4H)-benzopyran-4-one hydrochloride (LY294002; 50 µM) were added to the intracellular solution in the pipette to block NO production and release in the measured GnRH neuron. The pipette solution containing intracellularly applied drug was allowed to equilibrate with the intracellular milieu of the cell for 15 min before starting recording.

Time distribution graphs of frequencies were generated by using 1-min time bins to show time courses of effect of E2.

To show action of E2 onto the firing, resting potential (V_rest_), R_in_, and C_m_ in GnRH neurons of proestrous mice, current clamp measurements were conducted. Three 900-ms-long current steps were applied (-25, 0, and +25 pA). V_rest_ was evaluated from the 0-pA step, firing was analyzed during the depolarizing step. The R_in_ was determined from the voltage response to the application of hyperpolarizing current. The time constant was the time required to reach 63% of the maximum voltage response to hyperpolarizing current (Spergel et al., 1999). The C_m_ was then calculated by dividing the time constant by the R_in_. After control recording, E2 was pipetted into the measurement chamber and 1, 3, 5, and 10 min later, the three current steps were repeated.

### Loose patch measurements

Recording of action current firing of GnRH neurons was conducted at 33°C. Pipette potential was 0 mV, pipette resistance 1–2 MΩ, and resistance of loose-patch seal 7–40 MΩ. The pipette solution contained: 150 mM NaCl, 3.5 mM KCl, 2.5 mM CaCl_2_, 1.3 mM MgCl_2_, 10 mM HEPES, and 10 mM glucose (pH 7.3).

After recording basal action currents, the E2 (200 pM) was added in a single bolus to the brain slice in the recording chamber, and the recording continued for a subsequent 10 min, similarly to the method described in the whole-cell clamp measurements.

### Reagents and chemicals

GABA_A_-R blocker picrotoxin (100 μM, Sigma; Seidl et al., 2014; Keshavarzi et al., 2015); the wide-spectrum ionotropic Glu-R inhibitor kynurenic acid (2 mM, Sigma; Zheng et al., 2011; Ide et al., 2013); the AMPA/kainate receptor (AMPA/kainate-R) antagonist 2,3-dihydroxy-6-nitro-7-sulfamoyl-benzo[f]quinoxaline-2,3-dione (NBQX, 10 μM; Tocris; Keshavarzi et al., 2015; Lo et al., 2016); the ERβ antagonist PHTPP (1 µM; Tocris; Kajta et al., 2013; Saleh et al., 2013); the membrane-impermeable NO-scavenger CPTIO (1 mM, Sigma; Makara et al., 2007; Mironov and Langohr, 2007); E2 (200 pM, Sigma; Nelson et al., 1992; Christian et al., 2005; Freeman, 2006; Chu et al., 2009); the Src kinase inhibitor PP2 (10 µM; Sigma; Grishin et al., 2005); the PI3K inhibitor LY294002 (50 µM; Sigma; Zhang et al., 2016).

### Statistical analysis

For electrophysiology, each experimental group contained 8–12 recorded cells from six to nine animals. Recordings were stored and analyzed off-line. The mPSC frequency was calculated as number of spikes divided by the length of the respective period (5 min “control period” and 10 min “agonist period,” respectively). The effect of the treatments was displayed as a percentage ratio of the value of the respective mPSC parameter of the control and the agonist period. Each neuron served as its own control when drug effects were evaluated. Event detection was performed using the Clampfit module of the PClamp 10.4 software (Molecular Devices Co.). Group data were expressed as mean ± SEM. Statistical analyses were conducted using Prism 3.0 (GraphPad Software, Inc.) and Statistica 13.1 (Dell Software). Two-tailed Student’s *t* test, and one-way ANOVA test followed by Dunnett’s (ANOVA+D) *post hoc* test were applied for comparison of groups and the differences were considered as significant at *p* < 0.05. Cumulative probabilities of interevent intervals of each group were pooled (Liu et al., 2017) and analyzed by using Kolmogorov–Smirnov test to show statistical differences between the frequencies of the control and E2-treated neurons (Table 1).

### Animal preparation for triple-labeling immunocytochemistry

Mice (*n* = 4) were anaesthesized with a mixture of ketamine and xylazine (ketamine 50 mg/kg, xylazine 10 mg/kg body weight, i.p.) at 4:30 P.M. on the day of proestrus and were perfused transcardially with 10 ml 0.01 M PBS pH 7.4, followed by 50 ml 4% paraformaldehyde in 0.1 M phosphate buffer pH 7.4 (PB). The brains were rapidly removed and the brains were cryoprotected in 30% sucrose in 0.01 M PBS overnight at room temperature.

### Tissue preparation for triple-labeling immunofluorescence

Serial, 25-µm-thick coronal sections through the vascular organ of the lamina terminalis (VOLT) were cut on freezing microtome. The sections were pretreated first with 0.5% Triton X-100 and 0.5% H_2_O_2_ in 0.01 M PBS for 15 min. Nonspecific antibody binding was blocked with treatment in 2% normal horse serum (NHS) in PBS for 20 min.

### Triple-labeling immunofluorescence for GnRH, sGC, and vGLUT2 or vGAT

Sections were incubated in a primary antibody mix containing guinea-pig anti-GnRH serum at 1:5000 dilution (gift from E. Hrabovszky), rabbit anti-soluble guanylate cyclase α1 subunit (sGC) serum at 1:250 dilution (Sigma) and either sheep anti-vesicular glutamate transporter 2 (vGLUT2) serum at 1:1000 dilution (gift from M. Watanabe) or mouse anti-vGLUT2 serum at 1:1000 dilution (Millipore Bioscience Research Reagents) or sheep anti-vesicular GABA transporter (vGAT) serum at 1:5000 dilution (gift from M. Watanabe) in 2% NHS for 2 d at 4°C. Then, the sections were incubated in a mixture of Alexa Fluor 488-conjugated donkey anti guinea-pig IgG (1:250, Jackson), Dylight 405-conjugated donkey anti-sheep or mouse IgG (1:250, Jackson), and Alexa Fluor 555-conjugated donkey anti-rabbit IgG (1:500, Jackson) for 2 h, mounted onto glass slides, and cover slipped with Vectashield mounting medium (Vector).

Representative images were taken using Zeiss LSM 780 confocal microscope (Carl Zeiss GmbH) using line by line sequential scanning with laser excitation lines 410–483 nm for Dylight 405, 490–553 nm for Alexa Fluor 488, and 566–697 nm for Alexa Fluor 555; beam splitter/emission filters, main beam splitter (MSB) 405 nm for Alexa Fluor 405, MSB488/561 nm for Alexa Fluor 488, and Alexa Fluor 555. For 40× and 60× oil lenses, pinhole sizes were set to obtain optical slices of 2 and <1 μm in thickness, respectively, and the series of optical sections were recorded with 0.6-μm Z steps.

Images were analyzed with Zen 2012 (Carl Zeiss GmbH) and with Adobe Photoshop (Adobe System Inc.).

For the quantitative analyses, the images were taken using C2 Nikon confocal microscope (Nikon) using laser excitation lines 408 nm for Dylight 405, 488 nm for Alexa Fluor 488, and 561 nm for Alexa Fluor 555, dichroic mirror: 405(408)/457(440)/561/640(633); emission filters: 510/84 bandpass filter for Dylight 405, 593/40 bandpass filter for Alexa Fluor 488, and 635 long pass filter. Z-stack series of images were taken using a 60× oil immersion lens and the pinhole size was set to optimal for the lens. The series of the optical sections were recorded with 0.5-μm Z steps and the analyzed cells thickness were minimum 6 μm (>10 images). For the analyses we used orthogonal view in the NIS-elements Viewer 4.20 program and the colocalisation was accepted if it was seen in *XY*, *XZ*, and *YZ* planes. Juxtaposition was accepted if there was no visible gap between the GnRH-IR neuron and and the axon varicosity. Seven to nine sections were scanned from each animal (vGLUT2 *n* = 3; vGAT *n* = 4). A total of 1051 vGAT-IR varicosities were counted on the surface of 97 GnRH neurons, and 938 vGLUT2-IR varicosities were counted on the surface of 68 GnRH neurons.

The specificity of GnRH (Hrabovszky et al., 2011), sGC α1 (Szabadits et al., 2007), vGAT (Hrabovszky et al., 2012a), and vGLUT2 (Hrabovszky et al., 2012a) antibodies has been reported in the literature (for statistics, see Table 1).

**Table 1.** Statistical table

	Data structure	Type of test	Power
a	Normal distribution	Student’s *t* test	0.95
b	Normal distribution	One-way ANOVA with Dunnet’s *post hoc* test	0.95
c	Normal distribution	One-way ANOVA	0.95
d	Normal distribution	Kolmogorov–Smirnov	0.95

## Results

### High physiologic concentration of E2 increases firing rate in GnRH neurons in proestrus afternoon

**Figure 1.**
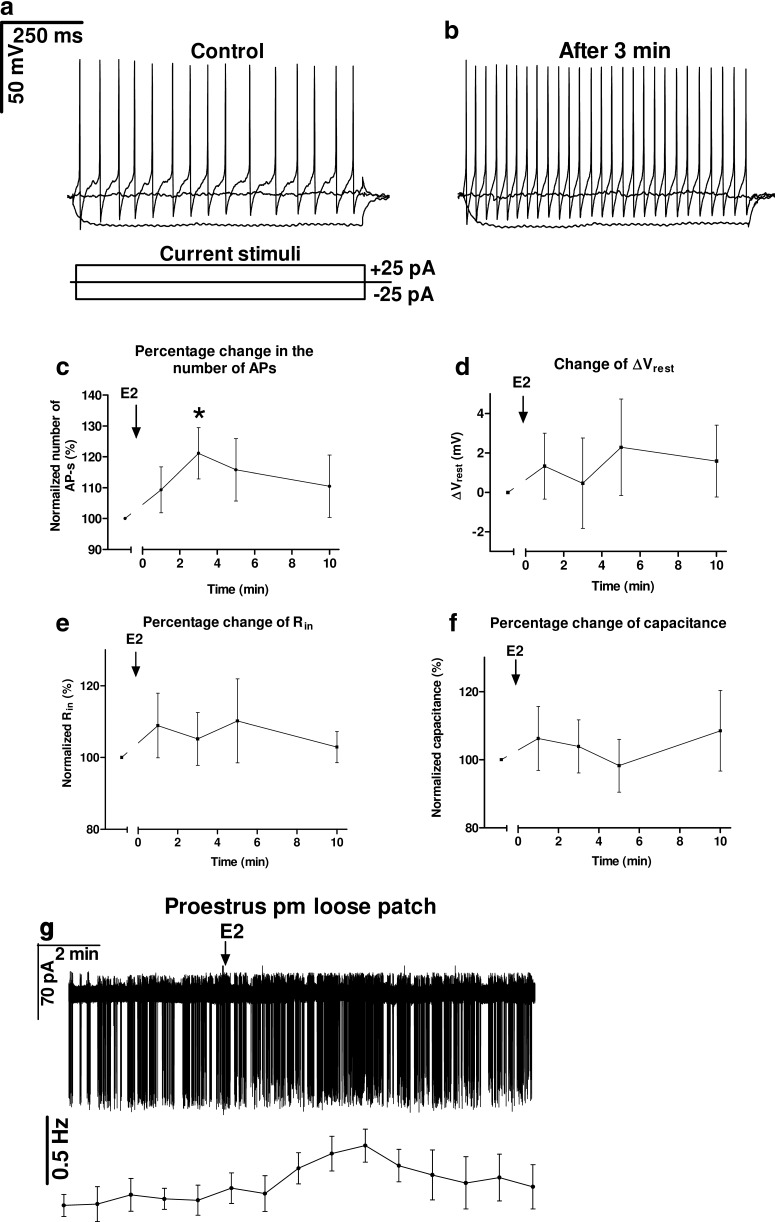
High physiologic concentration of E2 (200 pM) increases firing in GnRH neurons. ***A–C***, Number of APs elevated 3 min after E2 administration evoked by the depolarizing current step. ***D***, Changes in the resting potential showed no significant alteration. ***E***, R_in_ presented no E2-dependent change during the 10 min period examined. ***F***, Membrane capacitance also showed no change. ***G***, Loose patch clamp recording also confirmed the elevation of firing rate; *p < 0.05.

Plasma concentration of E2 in proestrus afternoon (proestrus P.M.) is in the range of a couple of hundred pM in rodents (Nelson et al., 1992; Freeman, 1994). In humans, the preovulatory level of E2 is also several hundred pM (Lin and Sun, 2005). Therefore, we administered E2 at 200 pM in our electrophysiological experiments, modeling the high physiologic dose characteristic for the proestrous afternoon stage of the gonadal cycle. Current clamp measurements revealed that 3 min after the E2 application the number of action potentials (APs) evoked by the depolarizing current injection increased significantly (121.14 ± 8.30% of the control value 19 ± 2.4 APs, *p* = 0.041^a^; Fig. 1*A–C*; Table 2). Other parameters, such as ΔV_rest_, R_in_, and C_m_ showed no change (Fig. 1*D–F*; Table 2). Loose patch recordings demonstrated similar increase in the firing rate (134 ± 12.1% of the control value 0.59 ± 0.08 Hz, seven neurons from four mice, Student’s *t* test, df = 6, *t* = 2.746, *p* = 0.0335^a^; Fig. 1*G*).

**Table 2.** Changes in firing, V_rest_, R_in_, and C_m_ in GnRH neurons on E2 administration

	Control value	Change (1 min)	Change (3 min)	Change (5 min)	Change (10 min)	*N*/*n*	df
Number of APs	19.5 ± 2.45	109.3 ± 7.4 %	121.1 ± 8.3 %	115.7 ± 10.1 %	110.5 ± 10.1 %	7/4	6
*t*		1.257	2.548	1.559	1.035		
*p* ^a^		0.2556	0.0436	0.1700	0.3405		
V_rest_	-53.6 ± 3.8 mV	1.33 ± 1.67 mV	0.46 ± 2.28 mV	2.29 ± 2.45 mV	1.59 ± 1.82 mV	7/4	6
*t*		0.7971	0.2037	0.9368	0.8751		
*p* ^a^		0.4558	0.8453	0.3850	0.4152		
R_in_	998.4 ± 127.8 MOhm	108.9 ± 9.0 %	105.2 ± 7.3 %	110.2 ± 11.7 %	102.9 ± 4.4 %	7/4	6
*t*		0.9903	0.7067	0.8739	0.6694		
*p* ^a^		0.3603	0.5063	0.4158	0.5281		
Capacitance	26.37 ± 1.1 pF	106.2 ± 9.4 %	103.9 ± 7.8 %	98.2 ± 7.7 %	108.5 ± 11.8 %	7/4	6
*t*		0.6632	0.4986	0.2269	0.7194		
*p* ^a^		0.5318	0.6358	0.8280	0.4990		

*N* = number of neurons measured. *n* = number of animals used for the given experiment.

df = degree of freedom of Student’s *t* test of percentage data where each neuron serves as its own control.

*t* = *t* values of Student’s *t* test of percentage data.

*p* = *p* probability values of Student’s *t* test of percentage data.

C_m_= cell capacitance.

### E2 increases frequency of mPSCs in GnRH neurons in proestrus afternoon

**Figure 2.**
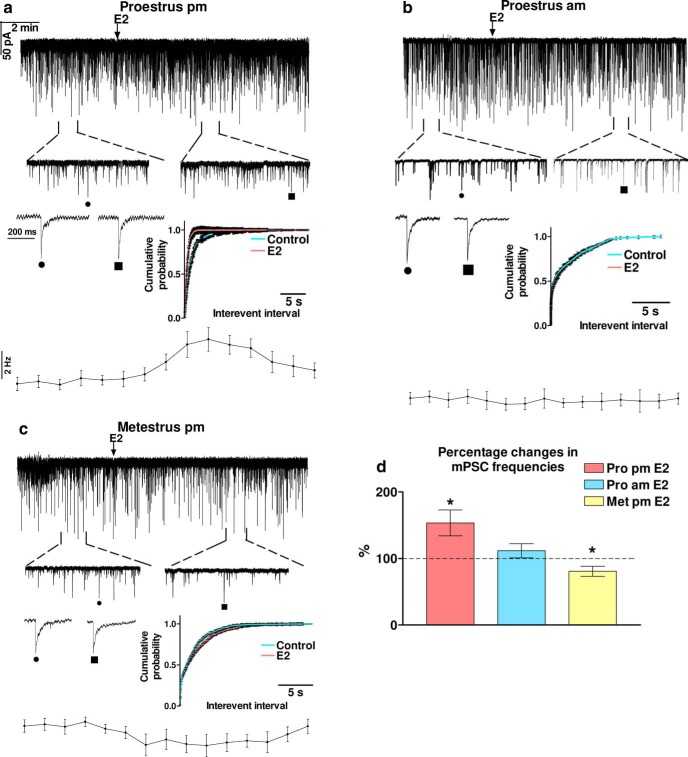
Effect of high physiologic concentration of E2 (200 pM) on the mPSCs of GnRH neurons in various phases of the estrous cycle. ***A***, E2 increased frequency of mPSCs in proestrus afternoon. ***B***, mPSC frequency showed no change when E2 was administered in proestrus morning. ***C***, E2 decreased mPSC frequency in metestrus afternoon. ***D***, Bar graph exhibits that E2 facilitated fast inputs to GnRH neurons in proestrus afternoon significantly but not in proestrus morning or in metestrus afternoon. The inserts below the 15 min recordings are 1-1 min zoomed periods from the respective recording before and after E2 administration. Individual PSC events are also shown from the control (to indentify in the 1-min zoomed periods, it is tagged by •) and the E2-treated (tagged by ▪) periods. Time course of the mean frequency and cumulative probabilities of the interevent intervals are presented under each recording; *p < 0.05.

Application of E2 (200 pM) resulted in a significant increase in the frequency of mPSCs (153.2 ± 17.33% of the control value 1.869 ± 0.1877 Hz) in GnRH neurons (Student’s *t* test, *p* = 0.0107^a^; Fig. 2*A*; Table 3). Elevation of the mPSC frequency started in 1-2 min after administration of E2 and slowly dampened after ∼10-min-long period of washout, as shown by the frequency distribution graph under the recording. Cumulative probability plots also demonstrated significant differences between the control and the treated interevent intervals (Kolmogorov–Smirnov, *p* = 0.0081^d^). In contrast, values of amplitude, rise, and decay τ of the mPSCs presented no significant change (Table 3). These results together suggested the involvement of a presynaptic process in the effect of E2 on the recorded mPSCs at late afternoon of proestrus. To examine whether the observed elevation in the mPSC frequency is specific for the time of killing, action of E2 on the mPSCs was investigated in the proestrus morning (proestrus A.M.). Using the same high physiologic dose of E2, we measured the parameters of the mPSCs of GnRH neurons. In contrast to the elevation observed at proestrus P.M., no significant alteration was detected in the mPSC frequency of GnRH neurons (Fig. 2*B*; Table 3**)**. Amplitude, rise, and decay τ parameters also showed no change (Table 3).

**Table 3.** Changes in mPSC parameters in GnRH neurons on E2 administration

Frequency of mPSCs	Frequency of control period (Hz)	Percentage change (%)	*N*/*n*	df	*t*	*p*
Pro pm E2	1.869 ± 0.1877	153.2 ± 17.33	12/7	11	3.067	0.0107^a^
Pro am E2	1.074 ± 0.2078	110.2 ± 8.58	12/8	11	1.189	0.2593^a^
Met pm E2	1.120 ± 0.2290	80.6 ± 7.54	9/6	8	2.591	0.0321^a^
Pro pm PHTPP+E2	2.059 ± 0.2281	93.4 ± 9.07	7/4	6	0.7243	0.4962^a^
Pro pm intraPHTPP+E2	1.790 ± 0.5129	97.1 ± 9.16	7/5	6	0.3222	0.7582^a^
Pro pm CPTIO+E2	2.006 ± 0.2569	106.1 ± 5.93	9/5	8	1.027	0.3346^a^
Pro pm PP2+E2	1.555 ± 0.2891	106.2 ± 6.06	8/6	7	1.014	0.3441^a^
Pro pm LY294002+E2	1.703 ± 0.1989	102.2 ± 5.88	8/6	7	0.3805	0.7148^a^
Pro pm Kynu+E2	1.279 ± 0.2357	133.7 ± 12.27	7/4	6	2.746	0.0335^a^
Pro pm Picro+E2	0.631 ± 0.1376	143.1 ± 17.23	7/4	6	2.492	0.0470^a^
Amplitude of mPSCs	Amplitude of control period (pA)	Percentage change (%)	*N*	df	*t*	*p*
Pro pm E2	-24.43 ± 2.067	100.7 ± 2.19	12/7	11	0.2760	0.7952^a^
Pro am E2	-36.41 ± 5.148	101.5 ± 1.15	12/8	11	1.343	0.2062^a^
Met pm E2	-34.51 ± 6.257	101.2 ± 2.84	9/6	8	0.4301	0.6785^a^
Pro pm PHTPP+E2	-26.48 ± 3.562	97.0 ± 2.19	7/4	6	1.368	0.2203^a^
Pro pm intraPHTPP+E2	-27.72 ± 8.614	99.1 ± 1.18	7/5	6	0.7337	0.4908^a^
Pro pm CPTIO+E2	-25.05 ± 2.062	102.7 ± 2.192	9/5	8	1.216	0.2585^a^
Pro pm PP2+E2	-26.46 ± 2.464	101.8 ± 3.84	8/6	7	0.4638	0.6569^a^
Pro pm LY294002+E2	-36.90 ± 4.897	100.7 ± 1.18	8/6	7	0.5812	0.5793^a^
Pro pm Kynu+E2	-36.83 ± 2.286	98.1 ± 2.37	7/4	6	0.7722	0.4693^a^
Pro pm Picro+E2	-19.64 ± 2.107	97.0 ± 2.58	7/4	6	1.162	0.2894^a^
Rise τ of mPSCs	Rise τ of control period (ms)	Percentage change (%)	*N*	df	*t*	*p*
Pro pm E2	3.941 ± 0.5402	138.8 ± 43.13	12/7	11	0.8985	0.3882^a^
Pro am E2	4.287 ± 0.1746	134.3 ± 20.29	12/8	11	1.688	0.1194^a^
Met pm E2	4.760 ± 0.6285	109.1 ± 16.41	9/6	8	0.5552	0.5939^a^
Pro pm PHTPP+E2	3.710 ± 0.2690	103.3 ± 14.27	7/4	6	0.2302	0.8256^a^
Pro pm intraPHTPP+E2	4.175 ± 0.9160	116.7 ± 24.64	7/5	6	0.6763	0.5241^a^
Pro pm CPTIO+E2	3.771 ± 0.4979	143.9 ± 44.24	9/5	8	1.611	0.1459^a^
Pro pm PP2+E2	3.970 ± 0.4466	99.1 ± 13.22	8/6	7	0.0657	0.9494^a^
Pro pm LY294002+E2	3.708 ± 0.2870	92.6 ± 9.09	8/6	7	0.818	0.4403^a^
Pro pm Kynu+E2	4.220 ± 0.4710	101.2 ± 14.99	7/4	6	0.0789	0.9396^a^
Pro pm Picro+E2	3.006 ± 0.1134	124.4 ± 27.99	7/4	6	0.8729	0.4163^a^
Decay τ of mPSCs	Decay τ of control period (ms)	Percentage change (%)	*N*	df	*t*	*p*
Pro pm E2	10.60 ± 2.416	137.2 ± 27.44	12/7	11	1.656	0.1259^a^
Pro am E2	13.04 ± 5.393	130.8 ± 24.20	12/8	11	1.272	0.2297^a^
Met pm E2	13.34 ± 1.805	141.4 ± 23.43	9/6	8	1.768	0.1150^a^
Pro pm PHTPP+E2	13.44 ± 3.977	105.3 ± 22.41	7/4	6	0.2359	0.8214^a^
Pro pm intraPHTPP+E2	11.83 ± 1.433	125.7 ± 16.45	7/5	6	1.562	0.1692^a^
Pro pm CPTIO+E2	13.62 ± 2.368	113.4 ± 22.79	9/5	8	0.5898	0.5716^a^
Pro pm PP2+E2	10.67 ± 1.033	94.5 ± 7.38	8/6	7	0.7455	0.4803^a^
Pro pm LY294002+E2	13.45 ± 2.559	97.3 ± 9.51	8/6	7	0.2853	0.7837^a^
Pro pm Kynu+E2	12.48 ± 1.152	103.5 ± 8.27	7/4	6	0.4269	0.6843^a^
Pro pm Picro+E2	5.62 ± 1.101	79.3 ± 21.57	7/4	6	0.9602	0.3740^a^

*N* = number of neurons measured. *n* = number of animals used for the given experiment.

df = degree of freedom of Student’s *t* test of percentage data where each neuron serves as its own control.

*t* = *t* values of Student’s *t* test of percentage data.

*p* = *p* probability values of Student’s *t* test of percentage data.

Pro = proestrus; Met = metestrus; am = morning; pm = afternoon.

The effect of E2 treatment on the mPCSs of GnRH neurons was also investigated in brain slices from metestrous mice killed in the late afternoon. Application of the same high physiologic concentration of E2, however, resulted in a significant decrease of mPSC frequency (80.6 ± 7.54% from 1.120 ± 0.2290 Hz, Student’s *t* test, *p* = 0.0321^a^; Fig. 2*C*; Table 3). Cumulative probability plots also demonstrate significant differences in the control and the treated intervals (Kolmogorov–Smirnov, *p* = 0.0332^d^). No change was observed in the amplitude, rise, and decay τ, respectively (Table 3).

Comparison of the percentage data on the frequency of mPSCs demonstrated that the effect of E2 at the late afternoon of proestrus is significantly different from its action during the proestrus morning and metestrus afternoon (ANOVA+D, *p* = 0.0017^b^; Fig. 2*D*; Table 4). Percentage data for amplitude, rise, and decay τ showed no significant differences (Table 4). In addition, mPSC frequencies in the control periods of the three groups presented significant differences (ANOVA+D, *p* = 0.0146^b^; Table 5), with the highest frequency in proestrus P.M. (Table 3), suggesting that intensity of the synaptic input to GnRH neurons is the strongest in proestrus P.M. Amplitude, rise, and decay τ data of the control periods measured in the three groups, however, exhibited no significant differences (Table 5).

**Table 4.** ANOVA+D analysis of percentage changes in frequency, amplitude, rise, and decay τ of mPSCs, respectively, measured in proestrus pm, proestrus am, and metestrus pm

	*F*	df	*p*		*p* (Dunnett’s *post hoc* test)
Frequency	7.9114	32	0.0017^b^	Pro pm vs Pro am	0.034^b^
				Pro pm vs Met pm	0.0009^b^
Amplitude	0.0539	32	0.9475^b^	-	-
Rise τ	0.2374	32	0.7901^b^	-	-
Decay τ	0.0494	32	0.9518^b^	-	-

df = degree of freedom of ANOVA test of percentage data.

*F* = *F* values of ANOVA test of percentage data.

*p* = *p* probability values of ANOVA or Dunnett’s test of percentage data.

Pro = proestrus; Met = metestrus; am = morning; pm = afternoon.

**Table 5.** ANOVA+D analysis of frequency, amplitude, rise, and decay τ values of the control periods of mPSCs, respectively, measured in proestrus pm, proestrus am, and metestrus pm

	*F*	df	*p*		*p* (Dunnett’s *post hoc* test)
Frequency	4.8850	32	0.0146^b^	Pro pm vs Pro am	0.015^b^
				Pro pm vs Met pm	0.036^b^
Amplitude	2.1160	32	0.1382^b^	-	-
Rise τ	0.7290	32	0.4907^b^	-	-
Decay τ	0.1574	32	0.8550^b^	-	-

df = degree of freedom of ANOVA test of percentage data.

*F* = *F* values of ANOVA test of percentage data.

*p* = *p* probability values of ANOVA or Dunnett’s test of percentage data.

Pro = proestrus; Met = metestrus; am = morning; pm = afternoon.

### The E2-evoked effect on mPSCs utilizes ERβ and activates nNOS via Src kinase/PI3K/Akt pathway in proestrus P.M.

**Figure 3.**
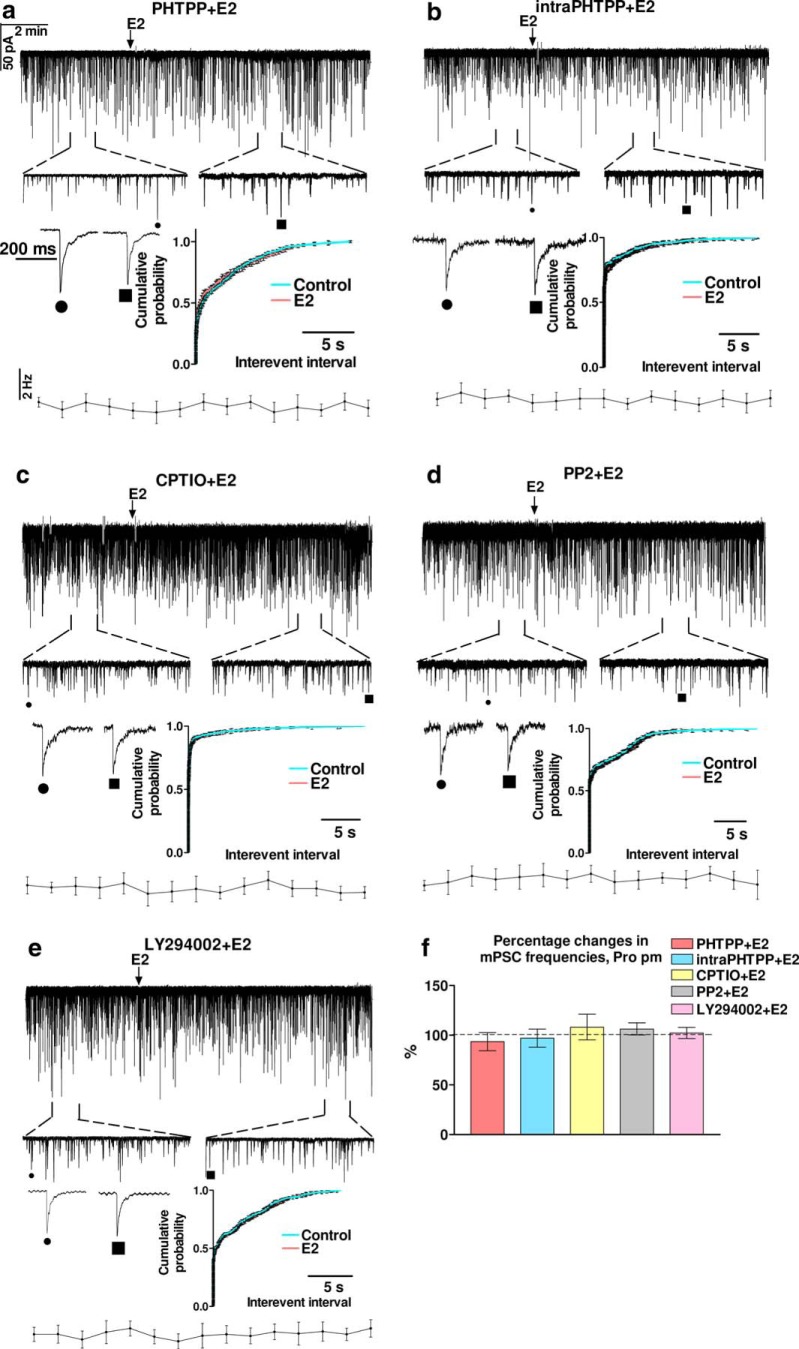
Effects of various blockers on the E2-evoked increase in the frequency of mPSCs in GnRH neurons in proestrus afternoon. The ERβ antagonist, PHTPP eliminated action of E2 in proestrus afternoon independently of whether PHTPP was applied extracellularly (***A***) or intracellularly (***B***). ***C***, Intracellular application of the NO-scavenger CPTIO abolished the effect of E2 in proestrus afternoon. ***D***, The Src inhibitor PP2 also eliminated the effect of E2. ***E***, The PI3K blocker LY294002 also abolished the action of E2. ***F***, Bar graph shows that effect of E2 was mediated via ERβ and the triggered retrograde NO signaling mechanism. The inserts below the 15 min recordings are 1-1 min zoomed periods from the respective recording before and after E2 administration. Individual PSC events are also shown from the control (to indentify in the 1-min zoomed periods, it is tagged by •) and the E2-treated (tagged by ▪) periods. Time course of the mean frequency and cumulative probabilities of the interevent intervals are presented under each recording.

To explore elements of the pathway activated by E2 (200 pM) in GnRH neurons in proestrus P.M., first we investigated the subtype and localization of the ER participating in the process. The main ER in GnRH neurons is the β-subtype (Hrabovszky et al., 2000, 2001, 2007; Kalló et al., 2001). In addition, the rapid action of E2 (1–2 min after application) on the mPSC frequency suggested the involvement of a non-genomic action. Therefore, the specific ERβ antagonist PHTPP was applied in the aCSF before E2 (200 pM) administration. The frequency of mPSCs presented no significant alteration (Fig. 3*A*,*F*; Table 3), suggesting that indeed ERβ was involved in the process. Amplitude, rise, and decay τ parameters also showed no significant change (Table 3).

Although PHTPP is lipophilic, therefore, its diffusion out of the cell cannot be excluded, intracellular administration provides more specific blockade than extracellular application of this drug. To increase the possibility that ERβ present in the recorded GnRH neuron participated in the observed effect of E2, PHTPP was applied intracellularly (intraPHTPP) in the recording patch pipette to block ERβ-mediated processes mostly in the recorded GnRH neuron. Intracellular administration of PHTPP also abolished the action of E2 (Fig. 3*B*,*F*; Table 3).

Previous studies have demonstrated (Farkas et al., 2016) that activation of the retrograde NO signaling pathway in the GnRH neurons results in increased mPSC frequency in GnRH neurons. To test the hypothesis that retrograde NO signaling mediates the effect of E2 on the synaptic input of the GnRH neurons, CPTIO, an NO-scavenger, was applied intracellularly in the recorded GnRH neuron before adding E2 to the slice. The mPSC measurements revealed that CPTIO fully eliminated action of E2 (Fig. 3*C*,*F*; Table 3).

As data from the literature indicate that in other hypothalamic cell types activation of ERβ may result in phosphorylation of nNOS and thus NO generation via an Src/PI3K/Akt-dependent manner (Gingerich and Krukoff, 2008), we applied the Src kinase inhibitor PP2 or the PI3K blocker LY294002 intracellularly in the pipette solution, separately. The mPSC measurements demonstrated that both PP2 and LY294002 fully abolished effect of E2 (Fig. 3*D–F*; Table 3).

Comparison of the effect of E2 in proestrus P.M. with the other five measurement groups involving the above described inhibitors (“Pro pm E2” vs “Pro pm PHTPP+E2,” “Pro pm intraPHTPP+E2,” “Pro pm CPTIO+E2,” “Pro pm PP2+E2,” and “Pro pm LY294002+E2,” respectively) revealed that the percentage change in frequency in Pro pm E2 differs significantly from each of the other five groups (ANOVA+D, *p* = 0.0026^b^; Table 6) demonstrating that indeed the ERβ/Src/PI3K/Akt/nNOS-dependent pathway was activated by E2 in proestrus P.M. Analysis has also revealed, that in the 5-min-long control periods Pro pm E2 data (Table 3) did not differ significantly from the other five groups (ANOVA^c^; Table 7) showing that none of the applied inhibitors evoked any change by themselves in the basal values of the analyzed parameters during the control periods.

**Table 6.** ANOVA+D analysis of the E2-triggered percentage changes in frequency, amplitude, rise, and decay τ of mPSCs, respectively, measured in proestrus pm, proestrus pm+PHTPP, proestrus pm+intraPHTPP, proestrus pm+CPTIO, proestrus pm+PP2, and proestrus pm+LY294002

	*F*	df	*p*		*p* (Dunnett’s *post hoc* test)
Frequency	4.343	50	0.0026^b^	Pro pm vs PHTPP	0.0033^b^
				Pro pm vs intraPHTPP	0.0060^b^
				Pro pm vs CPTIO	0.0151^b^
				Pro pm vs PP2	0.0201^b^
				Pro pm vs LY294002	0.0103^b^
Amplitude	0.6555	50	0.6589^b^	-	-
Rise τ	0.6889	50	0.6344^b^	-	-
Decay τ	0.8241	50	0.5392^b^	-	-

df = degree of freedom of ANOVA test of percentage data.

*F* = *F* values of ANOVA-test of percentage data.

*p* = *p* probability values of ANOVA or Dunnett’s test of percentage data.

Pro = proestrus; Met = metestrus; am = morning; pm = afternoon PHTPP = ERβ antagonist; intraPHTPP = intracellularly applied ERβ antagonist; CPTIO = NO-scavenger; PP2 = Src kinase inhibitor; LY294002 = PI3K inhibitor.

**Table 7.** ANOVA analysis of frequency, amplitude, rise, and decay τ values of the control periods of mPSCs, respectively, measured in proestrus pm, proestrus pm+PHTPP, proestrus pm+intraPHTPP, proestrus pm+CPTIO, proestrus pm+PP2, and proestrus pm+LY294002

	*F*	df	*p*
Frequency	0.4291	50	0.8259^c^
Amplitude	0.3411	50	0.8852^c^
Rise τ	0.1031	50	0.9910^c^
Decay τ	0.3504	50	0.8793^c^

df = degree of freedom of ANOVA test.

*F* = *F* values of ANOVA test.

*p* = *p* probability values of ANOVA test.

### Both GABAergic and glutamatergic inputs are detectable in proestrus P.M.

**Figure 4.**
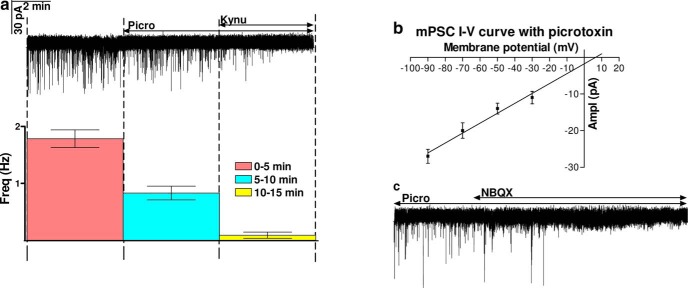
Both GABAergic and glutamatergic inputs are detectable during proestrus afternoon. ***A***, Picrotoxin decreased frequency of the mPSCs only partially. The residues were eliminated with the subsequent application of the ionotropic glutamate receptor blocker kynurenic acid. Average frequencies of the respective 5-5 min periods below the recording also showed this phenomenon. ***B***, I-V curve shows that reversal potential of the glutamate mPSCs (5.23 mV) is near the calculated value of that of glutamate channel (6.66 mV). ***C***, The AMPA/kainate-R inhibitor NBQX also abolished the residual mPSCs.

In female mice, solely the GABA_A_-R mediated component of the GABAergic transmission is detectable over GnRH neurons in coronal brain slices during the negative feedback period (Sullivan and Moenter, 2003; Balint et al., 2016). Nevertheless, there is only limited information about the role of GABA and glutamate in the regulation of GnRH neurons at proestrus P.M. Therefore, blocker of GABA_A_-R (picrotoxin) and two inhibitors of ionotropic glutamatergic transmission (kynurenic acid, NBQX) were probed in measuring mPSCs in GnRH neurons of brain slice from proestrous P.M. mice. Picrotoxin diminished the frequency of mPSCs but did not eliminate them fully (0–5 min: 1.784 ± 0.1549 Hz; 5–10 min: 0.831 ± 0.1211 Hz; Fig. 4*A*), because significant amount of mPSCs remained detectable in seven out of nine GnRH neurons (from six mice) after blocking the GABA_A_-Rs. Thus, the wide-spectrum ionotropic glutamatergic receptor blocker, kynurenic acid was added to the aCSF in the continuous presence of picrotoxin. Kynurenic acid fully abolished the residual mPSCs (10–15 min: 0.089 ± 0.0542 Hz; Fig. 4*A*), revealing the occurrence of strong glutamatergic neurotransmission beside the GABAergic one in proestrus P.M. Current-voltage relationship (I-V) of the mPSCs recorded in the presence of picrotoxin confirmed the contribution of ionotropic Glu-Rs because reversal potential of these mPSCs was 5.23 mV, which is rather near the calculated value of these glutamatergic ion channels (6.66 mV; Fig. 4*B*). To determine the subtype of the ionotropic Glu-R involved in the phenomenon, the AMPA/kainate-R blocker NBQX was added into the aCSF in the presence of picrotoxin. NBQX fully eliminated the glutamatergic mPSCs, suggesting involvement of AMPA/kainate-Rs (Fig. 4*C*).

### Both GABAergic and glutamatergic afferents to GnRH neurons express functional sGC receptors for NO signals

The natural question arises which synaptic input plays role in the NO-mediated elevated frequency of mPSCs observed on E2 application in proestrus P.M. To determine whether NO produced by GnRH neurons may target their incoming GABAergic and glutamatergic afferents via retrograde signaling, the presence of the NO receptor, sGC was studied in vGAT or vGLUT2-immunoreactive (IR) axon terminals contacting the cell membrane of GnRH neurons using triple-label immunofluorescence and confocal laser microscopy.

**Figure 5.**
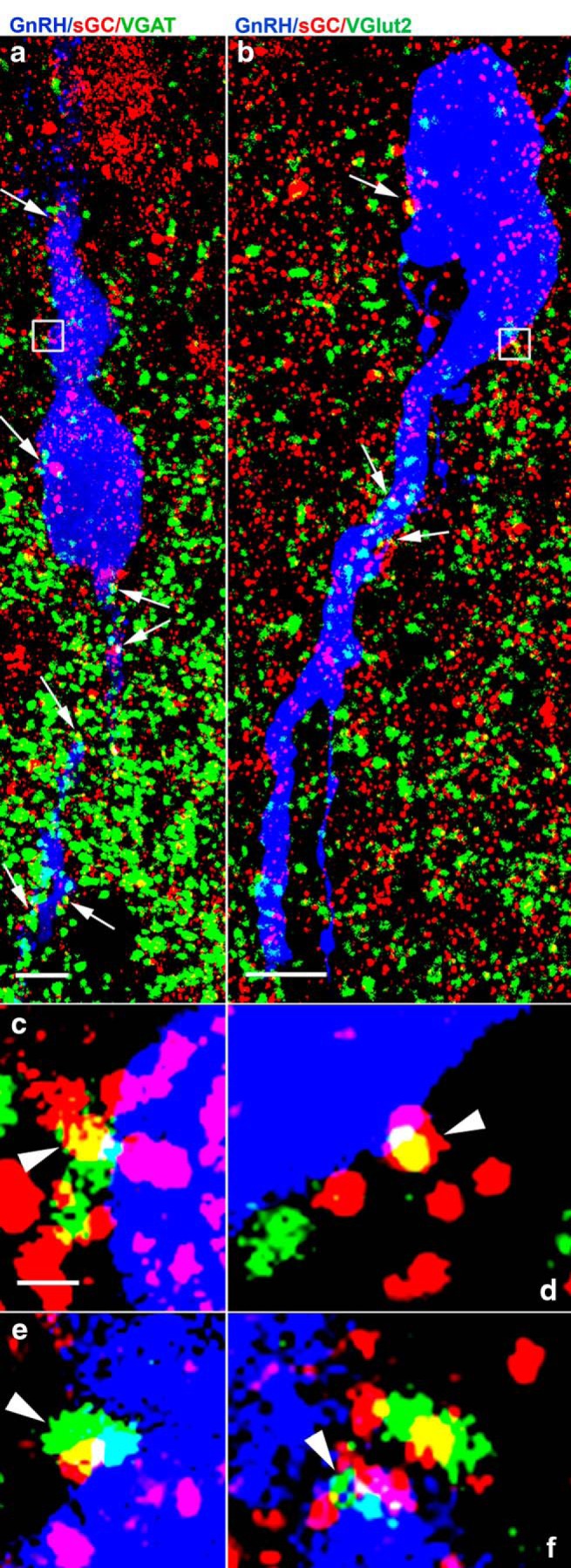
Presence of sGC-immunoreactivity in vGAT- (a, c, e) and vGLUT2-IR (***B***, ***D***, ***F***) axons contacting GnRH neurons of mice. Several vGAT-IR (green; ***A***, ***C***, ***E***), vGLUT2-IR (green; ***B***, ***D*, *F***), and sGC-IR (red) varicosities establish contact with the cell body and dendrites of GnRH neurons (blue). The yellow color in a population of vGAT-IR or vGLUT2-IR boutons in contact with the plasma membrane of GnRH neurons (arrows) demonstrates the presence of sGC in these profiles. The regions labeled with white rectangles are shown at higher power in ***C***, ***D***, arrowheads identify double-labeled varicosities. Further cases (***E***, ***F***) for demonstration of the expression of sGC immunoreactivity in vGAT-IR (***E***) and vGLUT2-IR (***F***) axon varicosities abutting GnRH-IR profiles. Scale bar = 5 µm, optical slice thickness = 0.6 µm.

Quantitative analyses of triple-labeled sections showed that double-labeled vGLUT2/sGC axon varicosities contacted 89.58 ± 4.9% of the analyzed GnRH-IR neurons (*n* = 3; 68 neurons) On the surface of the contacted neurons, 33.85 ± 5.08% of the vGLUT2 varicosities also contained sGC. Double-labeled vGAT/sGC axon varicosities were observed on the surface of 80.78 ± 5.55% GnRH-IR neurons (*n* = 4; 97 neurons). On the surface of contacted neurons, 37.95 ± 2.89% of the vGAT-IR axons also contained sGC (Fig. 5).

These data indicate that approximately third of the GABAergic and glutamatergic presynaptic afferents of GnRH neurons are sensitive to NO liberated from GnRH neurons, thus they may be the final targets of the estradiol-evoked actions.

**Figure 6.**
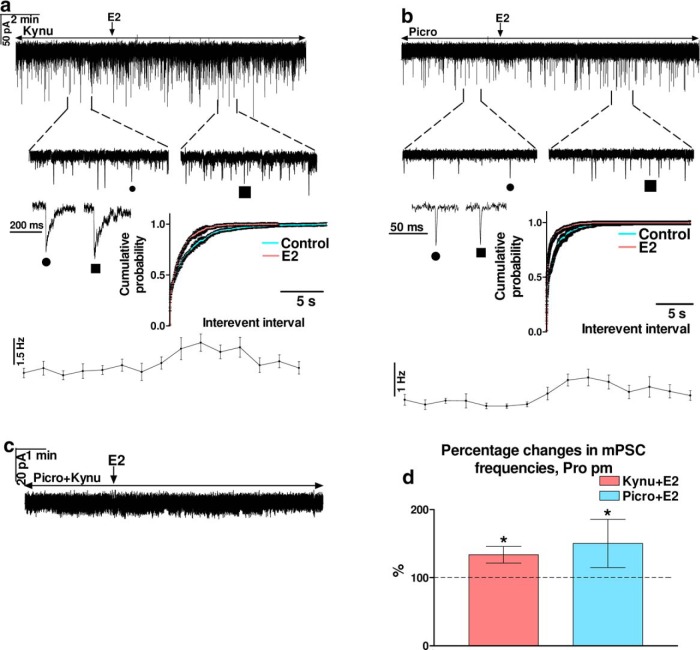
E2 (200 pM) acts on both fast GABAergic and glutamatergic (via AMPA/kainate-R) neurotransmissions to GnRH neurons in proestrus afternoon. ***A***, In the presence of kynurenic acid, E2 elevated frequency of the GABAergic mPSCs. ***B***, In the presence of picrotoxin, E2 increased frequency of glutamatergic mPSCs, too. ***C***, E2 was unable to trigger any mPSCs in the co-presence of picrotoxin and kynurenic acid. ***D***, Bar graph reveals that E2 increases frequency of both GABAergic and glutamatergic mPSCs significantly. The inserts below the 15 min recordings are 1-1 min zoomed periods from the respective recording before and after E2 administration. Individual PSC events are shown from the control (to indentify in the 1-min zoomed periods, it is tagged by •) and the E2-treated (tagged by ▪) periods. Time course of the mean frequency and cumulative probabilities of the interevent intervals are presented under each recording. Picro = picrotoxin, Kynu = kynurenic acid; *p < 0.05.

Presence of the sGC in both GABAergic and glutamatergic terminals contacting GnRH neurons were further investigated with mPSC measurements. E2 (200 pM) significantly increased the frequency of GABAergic mPSCs in the presence of kynurenic acid (Student’s *t* test, 133.7 ± 12.27%, *p* = 0.0335^a^; Fig. 6*A*,*D*), without affecting the amplitude, rise, and decay τ (Table 3). Time course of mean frequency under the recording shows that elevation started in 2-3 min and after reaching a peak it slowly decreased. The significant differences in the control and the treated interevent intervals are also confirmed by the cumulative probability plots (Kolmogorov–Smirnov, *p* = 0.0121^d^). E2 also elevated the frequency of glutamatergic mPSCs in the presence of picrotoxin (143.1 ± 17.23%, *p* = 0.0470^a^) with a time course similar to the one measured in the presence of kynurenic acid (Fig. 6*B*,*D*). The cumulative probabilities provided further evidence for the significant effect of E2 on the glutamatergic mPSCs (Kolmogorov–Smirnov, *p* = 0.0217^d^). No change in amplitude, rise, and decay τ were detected (Table 3). When both inputs were blocked simultaneously, no mPSCs were detected on E2 application (Fig. 6*C*), suggesting that other neurotransmitter systems were not influenced by the E2 administration. The measurements also showed that frequency (Student’s *t* test, *p* = 0.0352^a^), amplitude (*p* = 0.0001^a^), rise (*p* = 0.0276^a^), and decay τ (*p* = 0.0010^a^) values of the GABAergic mPSCs were significantly higher than those of glutamatergic ones measured during the control periods (Tables 3, 8).

**Table 8.** Student’s *t* test analysis of frequency, amplitude, rise, and decay τ values of mPSCs, respectively, measured in the presence of picrotoxin (detection of glutamatergic mPSCs) versus kynurenic acid (detection of GABAergic mPSCs)

	*t*	df	*p*
Frequency	2.373	12	0.0352^a^
Amplitude	5.529	12	0.0001^a^
Rise τ	2.506	12	0.0276^a^
Decay τ	4.307	12	0.0010^a^

df = degree of freedom of Student’s *t* test.

*t* = *t* values of *t* test.

*p* = *p* probability values of *t* test.

## Discussion

The present study provides electrophysiological and structural evidence for the involvement of ERβ and NO signaling in the mediation of the effect of E2 increasing fast neurotransmission onto GnRH neurons at late afternoon of proestrus. Accordingly, (1) E2 significantly increases the frequency of mPSCs in GnRH neurons; (2) this direct and rapid effect of E2 requires ERβ signaling in GnRH neurons; (3) the activation of ERβ signaling results in NO production via the Src/PI3K/Akt/nNOS-dependent pathway in GnRH neurons; and (4) the retrograde transmitter NO acts on both glutamatergic and GABAergic boutons conveying facilitatory tone to GnRH neurons and increases the excitatory fast neurotransmission.

### E2 increases frequency of mPSCs in GnRH neurons specifically in proestrus afternoon

Our present results showed that E2 at high physiologic concentration, which corresponds to the serum E2 level in proestrus afternoon, elevated mPSC frequency in GnRH neurons at late afternoon stage of proestrus, when the positive estradiol feedback culminates. This result is supported by Romanò et al. (2008), demonstrating an elevated GABAergic mPSC frequency in GnRH neurons on E2 treatment. E2 did not increase the frequency of mPSCs in proestrus morning or metestrus. Rather E2 even decreased the mPSC frequency in metestrus afternoon. This latter result is in good accordance with the literature data showing an E2-triggered attenuation of PSC frequency during metestrus (Balint et al., 2016). In contrast, a very recent paper published by Liu et al. (2017), has claimed that chronic E2 administration has no effect on GABA and glutamate inputs to GnRH neurons neither in negative nor in positive feedback periods, i.e., PSCs are independent from the stage of the estrous cycle (Liu et al., 2017). The most probable explanation to resolve this discrepancy is that two different experimental paradigms were used. In our model, intact late proestrous mice were studied with intact ovarian signaling mechanisms and physiologic concentration of E2 at the time of killing. The report from Liu et al. (2017) was based on the use of gonadectomized, estradiol replaced animals mimicking both the negative and positive feedback periods. Ovariectomy, in addition to ceasing the natural estradiol signaling, abolishes supply of numerous other indispensable hormones too, such as progesterone, activin, inhibin and anti-Müllerian hormone to the HPG axis including GnRH neurons (DeFazio and Moenter, 2002; Norwitz et al., 2002; Gregory and Kaiser, 2004; Sun and Moenter, 2010; Wang et al., 2010; Kenealy and Terasawa, 2012; Cimino et al., 2016; Stamatiades and Kaiser, 2017). In addition, the E2 replacement cannot mimic the natural fluctuation of E2 level.

Similar discrepancy exists between our present results revealing that robustly higher mPSC frequency was detected in proestrus P.M. than in proestrus A.M. or metestrus P.M., and those of Liu et al. (2017) finding no difference in the fast neurotransmission to GnRH neurons in positive versus negative feedback periods. The reason behind these contradictory data could be again the difference between the two models used. Nevertheless, indispensable role of the fast synaptic transmission in the effect of E2 on GnRH neurons has also been reported, since the E2-evoked changes in GnRH neuron firing were eliminated by blockade of GABAergic and glutamatergic neurotransmission (Christian and Moenter, 2008; Chu et al., 2009), in agreement with our present results.

Earlier studies have demonstrated that kisspeptin neuron populations of the AVPV region and the kisspeptin/neurokininB/dynorphin (KNDy) neurons of the arcuate nucleus (ARC) mediate the positive and negative feedback actions of E2 toward the GnRH system (Smith et al., 2005; Kalló et al., 2012; Clarkson et al., 2017; Lunenfeld and Buhler, 2017; Vastagh and Liposits, 2017). Human mutations and knock out animals clearly showed the importance of these indirect pathways in the feedback regulation of GnRH neurons (Mayer and Boehm, 2011; Brioude et al., 2013; Ratnasabapathy and Dhillo, 2013; Demirbilek et al., 2015). In contrast, our present data revealed that GnRH neurons are targets of the direct action of E2 during the positive feedback period. In addition, a very recent paper also demonstrated direct E2 action on mPSCs measured in GnRH neurons during the negative feedback period (Balint et al., 2016). However, these data seems to contradict with the critical importance of the indirect pathways. This discrepancy can be resolved if we hypothesize that the effect of E2 to facilitate the inputs of GnRH neurons during the afternoon of proestrus amplifies the effects of the indirect pathways that mediate the E2 positive feedback effect, while the inhibitory action of E2 on the inputs of GnRH neurons may prevent the influence of these pathways suggesting that the direct and indirect E2 effects work in concert to regulate GnRH neurons. This idea, however, requires further elucidation.

### The execution of direct, rapid effect of E2 requires functional ERβ in GnRH neurons

It is a well-known fact that GnRH neurons bear ERβ (Hrabovszky et al., 2000, 2001, 2007). Our present results clearly demonstrated that in proestrus P.M. these neurons responded to E2 via ERβ because PHTPP, the selective antagonist of this ER subtype, eliminated action of E2 on mPSCs. The intracellular application of this antagonist evoked similar blockade indicating that action of E2 is direct on ERβ in GnRH neurons. In addition, effect of E2 was rapid, starting in 1–2 min, suggesting a non-genomic action of this hormone. Similar rapid actions of E2 have already been explored in GnRH neurons, suggesting activation of intracellular signaling pathways via membrane-associated ERs (Abrahám et al., 2003; Cheong et al., 2012; Kwakowsky et al., 2014).

### Retrograde NO signaling is involved in the E2-triggered increase of mPSC frequency in GnRH neurons

The present results revealed a key role of the retrograde NO signaling pathway in mediation of the effect of E2 to presynaptic GABAergic and glutamatergic inputs of GnRH neurons. Presence of the functional nNOS has been recently shown in GnRH neurons, and the release of NO resulted in an elevated GABAergic mPSC frequency in these neurons (Farkas et al., 2016) via the retrograde NO signaling pathway. A very recent paper has also proved the presence of nNOS in GnRH neurons of ewes (Bedenbaugh et al., 2017). In addition, evidence was presented that postsynaptic actions such as binding of glucagon-like peptide-1 to its receptor in GnRH neurons could activate NO synthesis eventually leading to an increased mPSC frequency via this retrograde pathway (Farkas et al., 2016). In the present work, we found a similar scenario, since binding of E2 to ERβ could activate nNOS in GnRH neurons at late proestrus afternoon and the released NO did modulate the release probability of the GABA and glutamate neurotransmission from the afferent terminals. Fundamental role of NO in the regulation of the hypothalamic centers of reproduction has also been demonstrated *in vivo*, when L-NAME, a NOS inhibitor was infused into the preoptic region of female mice. The abolishment of NO signaling disrupted the estrous cycle and eventually resulted in infertility (d'Anglemont de Tassigny et al., 2007). Now, we have revealed not only the significance of NO in the function of GnRH neurons during positive E2-feedback period, but also showed the involvement of Src kinase and PI3K as crucial elements in the Akt-regulated phosphorylation of nNOS linked to the E2-activated ERβ.

It is intriguing that estradiol has completely opposite effects on the inputs of GnRH neurons during metestrus and proestrus afternoon. While the inhibitory effect is mediated by endocannabinoids during metestrus (Balint et al., 2016), the facilitatory effect is mediated by NO during the afternoon of proestrus. Further studies are needed to understand whether GnRH neurons use different retrograde transmitters in response to E2 during the different phases of the estrous cycle or the sensitivity of the inputs of GnRH neurons to the different retrograde transmitters is altered by the stages of estrous cycle.

### Both GABAergic and glutamatergic neuronal inputs are affected by E2

Although ionotropic glutamatergic mPSCs have already been demonstrated in GnRH neurons of sagittal slices of the mouse brain (Christian et al., 2009; Liu et al., 2017), these PSCs could have been recorded in GnRH neurons of acute coronal slices neither from male nor from female mice in negative feedback period, so far (Sullivan and Moenter, 2003; Farkas et al., 2010). In our present work, this excitatory synaptic transmission has been easily detected in the majority of GnRH neurons suggesting an altered, putatively increased, glutamatergic input to these cells during proestrus. This result partially explains the significantly higher mPSC frequency in proestrus P.M. versus that of in metestrus, described above. Since firing activity and PSC frequency correlate positively (Chu and Moenter, 2005; Christian and Moenter, 2007; Farkas et al., 2013) and firing rate is higher in positive than in negative feedback period (Silveira et al., 2017) these phenomena support further our present data.

It was an intriguing question, which synaptic inputs were affected by the effect of E2. Our present electrophysiological data revealed that both GABAergic and glutamatergic (presumably via AMPA/kainate-R) neurotransmission are influenced by E2 in proestrus P.M. This observation has also been confirmed at the structural level demonstrating the presence of sGC, the receptor of NO, in both types of axon terminals contacting GnRH neurons. Therefore, the NO released from GnRH neurons after activation of ERβ at late proestrus afternoon can reach both types of terminals and influence the release of GABA and glutamate from them.

Our present results indicate that there is an increased excitatory input to GnRH neurons in the proestrus afternoon before applying E2. It raises the possibility that this phenomenon is due to the high level of E2 remained in the brain slice after decapitation. The ANOVA analysis of the control period of recordings with various blockers presented in the Table 7, however, demonstrates that these control periods show no significant differences, regardless which inhibitor (ERβ antagonist or signaling cascade blockers) were applied. Therefore, we suggest that this difference is not due to acute E2 effect, but rather it is a genomic action of E2 that was exerted on GnRH neurons and/or on their inputs before the slice preparation or it is an estrogen independent effect.

**Figure 7.**
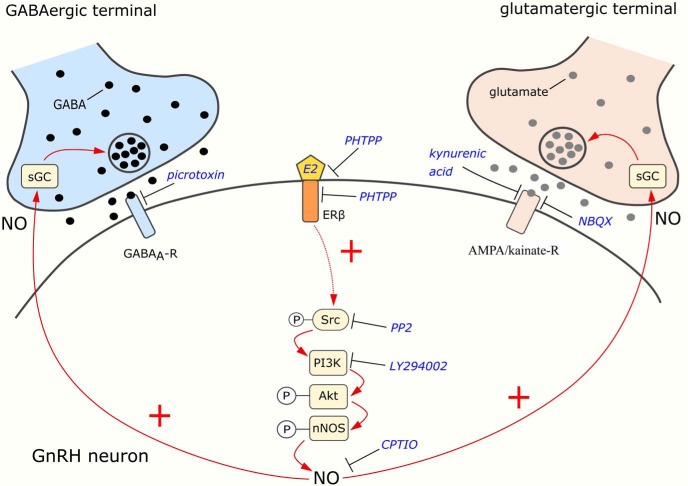
Schematic illustration of E2-ERβ signaling in GnRH neurons at proestrus afternoon and its proposed action on presynaptic GABA and glutamate release. E2 activates the phosphorylation of neuronal NO synthase (nNOS) via ERβ and the Src/PI3K/Akt signal transduction pathway resulting in increased NO production. NO then binds to its receptor, sGC, which is present in both GABAergic and glutamatergic afferent boutons of GnRH neurons. The process increases the release of GABA and glutamate that facilitates the activity of GnRH neurons via GABA_A_-R and AMPA/kainate-R.

To sum up, the proposed model of the rapid E2 action on GnRH neurons during the positive feedback period is illustrated in Figure 7. This study suggests that E2 binds to ERβ and triggers the synthesis and release of NO from GnRH neurons. Then, using the retrograde NO-signaling pathway, NO binds to sGC located in the presynaptic terminals of GABAergic and glutamatergic afferents, which eventually evokes an increased release of both GABA and glutamate into the synaptic cleft and in turn, excite GnRH neurons via GABA_A_ and AMPA/kainate receptors making GnRH neurons more sensitive to the inputs arriving from the AVPV and/or the Arc. The elucidation of the putative participation of the ERβ-NO signaling mechanism in GnRH neurons of humans during the preovulatory positive E2 feedback action raises a further challenge.

## Synthesis

Reviewing Editor: Quentin Pittman, University of Calgary Decisions are customarily a result of the Reviewing Editor and the peer reviewers coming together and discussing their recommendations until a consensus is reached. When revisions are invited, a fact-based synthesis statement explaining their decision and outlining what is needed to prepare a revision will be listed below. The following reviewer(s) agreed to reveal their identity: Martin Kelly.

Both reviewers and I agree that your study makes a contribution to the literature and in particular provides additional clarification over that from a recent publication where slightly different methodology was used (ovariectomy and E2 administration, vs natural cycling mice). Moreover, after discussion among us we agree that a revision would acceptable if you could adequately address the following points:

1. Clarify the actual dose of E2, as it is impossible to know the actual concentration given your description. In addition it would be useful to show a time course or its action that clearly depicts onset of the E2 effects and washout if any from a representative group of cells ( ie mean +/- SEM).

2. Given the reversed GABAergic PSPS due to the high intracellular chloride what is the ultimate effect on excitability? If both GABA and Glut inputs are augmented what is the overall effect? Thus the cell attached experiments suggested by one of the reviewers appears critical.

3. It is important to provide more information concerning the immuno and co-localization. How many times was this experiment repeated? In how many animals and how many sections? How many terminals were counted; some quantitation is definitely required here.

4. While we understand that statistics were calculated on cell numbers, please indicate how many animals were used for each experiment. This value could be added to the tables.

The individual reviews are appended below. They each contain additional suggestions that you may wish to consider but they need not all be addressed for the manuscript to be acceptable. However, where additional information or corrections are suggested these should be dealt with as outlined.

Reviewer 1:

This manuscript investigates the changes in miniature synaptic input to GnRH neurons across the estrous cycle as well as the changes in responsiveness of GnRH neurons to exogenous estradiol application across the estrous cycle. The authors report differences in the basal rate of miniature postsynaptic currents across different stages of the estrous cycle. They also report that acute application of 200pM estradiol induces an increase in the frequency of miniature synaptic currents recorded from GnRH neurons in brain slices taken from female mice on the afternoon of proestrous but not from mice on the morning of proestrous. The same protocol resulted in a reduction in the frequency of miniature postsynaptic currents in brain slices taken from metestrous mice. The authors then go on to show that blocking ERβ/Src/PI3K/Akt/nNOS signaling can prevent the estradiol induced enhancement of miniature postsynaptic current frequency. In support of nitric oxide signaling at synapses onto GnRH neurons, the authors present immunohistochemistry images showing colocalisation of soluble guanylyl cyclase and presynaptic GABA and glutamate bouton markers.

This is a timely study considering recent work (also published in eNeuro) has investigated changes in miniature synaptic current frequency across the estrous cycle and found no significant differences (Liu et al 2017). However, there are important methodological differences between the current and the Liu et al study which may explain the differing results.

Generally the study was well executed. However, there were several parts of the manuscript were details were missing or analysis was lacking. These and other comments are detailed below:

1) Methodology concerning application of estradiol not explained.

For example, it is stated that estradiol was added directly to the recording bath- What volume of 200pM estradiol ACSF solution was added and how long was this added for? Secondly, what was the volume of the tissue bath and the flow rate of the perfusion system? Based on these parameters, please report the estimated final concentration of estradiol in the tissue bath.

2) Latency and duration of estradiol effect not analyzed or reported.

It is mentioned in the text that the effect of estradiol on mPSC frequency started after 1-2 min, but no data is reported to support this statement. Please show a mean time course of mPSC frequency (in 1-minute time bins) for the 5 minutes prior and 10 minutes following estradiol application. This will allow the reader to assess both the average time course and the average magnitude of the estradiol effect.

3) Details and analysis of immunohistochemistry not reported.

Many aspects of the immunohistochemistry experiment are not reported. For example, the

estrous cycle stage and time of day the tissue was collected. Details of anaesthetic and perfusion fixation procedure are also not reported.

Was this immunohistochemistry only performed on one animal?

I do not think conclusions can be drawn from this experiment in its current form. To conclusively say that sGC is co-expressed in both VGlut and VGAT terminals, the authors must perform this immunohistochemistry on sections from multiple animals. More importantly, the colocalisation must be quantified.

4) Clarification of confocal imaging method required.

For example- the laser lines are reported as large ranges of wavelengths, however, for a laser, the light is normally of a very narrow wavelength. Secondly, the emission filters for the dyes don't make sense. Perhaps the authors have the numbers for laser lines and emission filters mixed up? Finally, what does MSB stand for?

5) In the discussion the authors frequency refer to Herbison et al when the correct citation is in fact Liu et al.

6) The authors should cite the paper below and note that it has also shown increases in GABA synaptic currents in GnRH neurons following acute applications of estradiol:

Romano N, Lee K, Abraham IM, Jasoni CL, Herbison AE (2008). Nonclassical estrogen modulation of presynaptic GABA terminals modulates calcium dynamics in gonadotropin-releasing hormone neurons. Endocrinology.

Reviewer 2:

The manuscript entitled “Estradiol increases glutamate and GABA neurotransmission into GnRH neurons....” counters a recent eNeuro (2017) paper from Herbison lab (Liu 2017, eNeuro 0259-17.2017) that reported there were no differences in the ionotropic GABA and glutamate input to GnRH neurons in an E2-treated, ovariectomized female “surge” model. The current paper uses intact cycling females on the afternoon of proestrus and contrast these females with the morning of proestrus in which bath applied E2 did not alter synaptic input and with metestrus females in which bath applied E2 inhibited the frequency of mPSCs. Although the investigators argue that this is a more physiological model to investigate the rapid effects of 17β-estradiol on modulating the synaptic activity, they do not address the fact that they are adding exogenous E2 on top of high circulating levels of E2. The downstream signaling effects of late proestrous levels of the gonadal steroid should persist into the in vitro experimental period. Therefore, the investigators need to control for the endogenous synaptic activity generated by circulating E2:

1) What are the effects of bath perfused 200 pM E2 on the resting membrane potential, input resistance and cell capacitance? The authors should report these parameters. Also, a 50 pA holding current is awfully large for these high input resistance GnRH neurons and would be indicative of a leaky membrane.

2) The results that are presented are minimal and for the most part in tabular form. The authors need to show cumulative probability plots and representative event traces in which the kinetics can be analyzed (e.g., Liu 2017, eNeuro 0259-17.2017).

3) The results depicted in Figure 3 would indicate that there is increased excitatory input to GnRH neurons on the afternoon of proestrus without bath applying E2. If this is an E2 generated phenomena, then the authors should be able to block this activity with the ERβ antagonist or with one of the signaling cascade blockers.

4) Based on the pharmacological blockade with picrotoxin and NBQX the authors conclude that there is a combination of GABAergic and glutamatergic excitatory input to GnRH neurons during the afternoon of proestrous. But the high chloride internal solution would augment the GABAergic inputs and bias the relative contribution of GABAergic versus glutamatergic input depicted in Figure 3. Why did the authors not use a standard K-Gluconate internal solution?

5) Also, Figure 3 appears to be one cell experiment. There are no standard error bars. The authors should show the group data.

6) PHTPP is lipophilic so dialyzing the cell with the ERβ antagonist does not ensure that it does not diffuse out of the cell and into the extracellular environment to affect synaptic activity.

7) An earlier publication by Balint et al. (Frontiers in Cellular Neuroscience 2016) argued for a CB1 receptor mediated suppression of GABAergic input to GnRH neurons in metestrous females. Is this mechanism not operable in proestrous females? What are the effects of blocking endocannabinoid signaling in proestrous females on the ionotropic synaptic input?

8) What is the functional output of the increased excitatory input to GnRH neurons? The authors should show in cell attached recordings that the bath perfusion of E2 increases the firing activity of GnRH neurons based on robust (153%) increase in synaptic activity.

Minor Comments:

1. Some editing of the text is needed: line 10, “a high physiological dose of estradiol.....'

2. line 267, “Previous studies have demonstrated... (Farkas et al., 2016).”

3. Line 358, “the most probably explanation to resolve.....”

4. Line 390, “This discrepancy can be resolved...”
